# Assessment of the Antimicrobial Activity of Olive Leaf Extract Against Foodborne Bacterial Pathogens

**DOI:** 10.3389/fmicb.2017.00113

**Published:** 2017-02-02

**Authors:** Yanhong Liu, Lindsay C. McKeever, Nasir S. A. Malik

**Affiliations:** Molecular Characterization of Foodborne Pathogens Research Unit, Eastern Regional Research Center, Agricultural Research Service, U.S. Department of Agriculture, WyndmoorPA, USA

**Keywords:** *L. monocytogenes*, olive leaf extract, bacterial growth inhibition, biofilm formation, natural antimicrobials

## Abstract

Olive leaf extract (OLE) has been used traditionally as a herbal supplement since it contains polyphenolic compounds with beneficial properties ranging from increasing energy levels, lowering blood pressure, and supporting the cardiovascular and immune systems. In addition to the beneficial effects on human health, OLE also has antimicrobial properties. The aim of this work was to investigate the antimicrobial effect of OLE against major foodborne pathogens, including *Listeria monocytogenes, Escherichia coli* O157:H7, and *Salmonella* Enteritidis. Our results demonstrated that at a concentration of 62.5 mg/ml, OLE almost completely inhibited the growth of these three pathogens. In addition, OLE also reduced cell motility in *L. monocytogenes*, which correlated with the absence of flagella as shown by scanning electron microscopy. Moreover, OLE inhibited biofilm formation in *L. monocytogenes* and *S*. Enteritidis. Taken together, OLE, as a natural product, has the potential to be used as an antimicrobial to control foodborne pathogens.

## Introduction

Foodborne pathogens can cause large outbreaks associated with a variety of foods, which is a global concern with significant impact on human health. According to a recent report from the Centers for Disease Control and Prevention (2011), it is estimated that 1 in 6 Americans get sick from contaminated foods and 3,000 people die from foodborne diseases annually^1^. Therefore, control of foodborne pathogens is a major concern for regulatory agencies and the food industry. Three important foodborne pathogens include *Escherichia coli* O157:H7, *Listeria monocytogenes*, and *Salmonella* enterica.

One of the means in which foodborne pathogens can be controlled is through the use of food preservatives, which can be classified as synthetic chemical and natural antimicrobial compounds. Compared to synthetic food preservatives, plant antimicrobials attract more attention since they are generally recognized as safe, and may benefit to human health (Seow et al., 2014). Moreover, plant antimicrobials may also add flavor foods. There have been some extensive searches for potential natural antimicrobials with a broad spectrum of antimicrobial activities that can be used to extend the shelf life of perishable foods (Calo et al., 2015). Some potentially useful plant antimicrobials have been identified (Friedman et al., 2009, 2013; Ravishankar et al., 2010). The emerging antibiotic resistance concerns and the consumers’ preference for natural food preservatives raises the necessity to continually look for a plant antimicrobial for the food industry.

Olive leaf extract (OLE) can be considered a plant antimicrobial with both antimicrobial and antioxidant activities (Lee and Lee, 2010). OLE also has health benefits such as increasing energy levels, lowering blood pressure, and supporting the cardiovascular and immune systems (Khayyal et al., 2002; Visioli and Galli, 2002; Covas, 2007; El and Karakaya, 2009). OLE has been shown to have antimicrobial activities against foodborne pathogens such as *Staphylococcus aureus, E. coli, Salmonella* spp., and *L. monocytogenes* (Techathuvanan et al., 2014). For example, OLE has been used to reduce bacteria in shrimp and organic leafy greens (Moore et al., 2011; Ahmed et al., 2014). In addition, OLE has been shown to enhance the quality and shelf-life of meat products (Hayes et al., 2010a,b). Despite the broad spectra of antimicrobial activities of OLE, the mode of its action on foodborne pathogens is still unclear.

The purpose of this study was to investigate how OLE affects the growth and function of *L. monocytogenes*, as well as other foodborne pathogens. The anti-bacterial mechanism of OLE on *L. monocytogenes* was also investigated. The ultimate goal was to determine if OLE is a potential antimicrobial for use in the food industry, as either a food additive or sanitizing material for the processing plants.

## Materials and Methods

### Olive Leaf Extraction and High Performance Liquid Chromatography (HPLC) Analysis

Polyphenols from olive leaves, and commercial OLE products purchased from GNC health stores (Pittsburg, PA, USA), were extracted by the established extraction methods in our laboratory (Malik and Bradford, 2006). Briefly, the fresh olive leaves were immediately frozen and stored at -80°C, and 6.25-g portions of frozen leaf samples were pulverized in liquid nitrogen and then extracted in 25 ml of 80% ethanol. Commercial OLE samples were directly poured in 80% ethanol for extraction as described previously (Malik and Bradford, 2006, 2008).

The separation and identification of polyphenols in extracts were performed as described previously (Malik and Bradford, 2006, 2008). In brief, an aliquot of OLE passed through a 20-μm filter was used for reversed phase high performance liquid chromatography (HPLC) analysis using Waters Symmetry C_18_ (5 μm particle) column (3.9 mm × 150 mm) maintained at 35°C. The column was eluted with a gradient solvent system comprising of 100% acetonitrile (solvent A) and 0.02% trifluoroacetic acid (solvent B) at a flow rate of 1ml/min. The starting composition of the gradient was 5% A and 95% B that was linearly increased to 10 % A in 10 min. After 10 min solvent A was increased to 30% in 24 min and thereafter to 40% in 11 min. The column was washed with 80%A and then equilibrated to 95% B for 10 min before each run. The elution profiles were detected at 280 nm and the major peaks were identified by comparison of retention times with standard compounds and also the UV spectra of the peak.

### Bacterial Inhibition Assays

Three bacterial strains (*L. monocytogenes* F2365, *E. coli* O157:H7, and *S.* Enteritidis) used in this study were from the Eastern Regional Research Center (ERRC) culture collection. Single colonies of *L. monocytogenes* F2365, *E. coli* O157:H7 and *S.* Enteritidis were inoculated in 5 ml of Brain Heart Infusion (BHI) broth (Sigma–Aldrich Inc., St. Louis, MO, USA) and incubated at 37°C overnight with agitation at 200 rpm. Bacterial inhibition assays were performed using a two-fold dilution method (Balouiri et al., 2016). Briefly, 75 μL of a two-fold serially diluted OLE (250 mg/ml stock) were placed in a 100-well plate together with 225 μL 1:100 diluted bacterial overnight culture. The 100-well plate was incubated in a Bioscreen C (Oy Growth Curves AB Ltd., Helsinki, Finland) at 37°C for 24 h with OD600 readings recorded every hour. The OLE without bacteria was used as a negative control. The bacteria without OLE were used as positive controls. Percent growth inhibition was calculated using the following equation: Growth inhibition (%) = OD_(bacteria)_–{OD_(bacteria+Extract)_ – OD_(Extract)_} / OD_(bacteria)_ × 100, where OD_(bacteria)_ is the O.D._600 nm_ for the positive control, OD_(bacteria+Extract)_ is the O.D._600 nm_ for the sample treated with OLE, and OD_(bacteria)_ is the O.D._600 nm_ for the negative control. Each growth experiment was repeated three times.

The oleuropein and vabascoside were obtained from Alkemist Labs (Costa Mesa, CA, USA). Each compound was dissolved at a concentration of 100 mg/ml with 80% methanol. The methanol was removed via rotary evaporation for 30 min at 40°C and the concentration was brought back to 100 mg/ml with sterile distilled water. 80% methanol was also rotovapped to be used as a control alongside the compounds, to confirm that any remaining alcohol had no effect on bacterial growth. The compounds were diluted using the serial two-fold serial dilution method described above.

### Bacterial Motility Assays

A single colony of *L. monocytogenes* F2365 was inoculated in 5 ml of BHI broth and incubated at 37°C overnight with agitation at 200 rpm. Bacterial overnight cultures (1.5 μL) were injected into three spots on freshly prepared BHI agar plates (0.3% agar) supplemented with different concentrations (1.95, 3.9, and 7.8 mg/ml) of OLE. The plates were incubated at room temperature for 48 h. The diameters of the bacterial growth rings were measured. Each experiment was repeated three times.

### Scanning Electron Microscopy

A single colony of *L. monocytogenes* F2365 was inoculated in 5 ml of BHI broth supplemented with different concentrations (7.8, 31.3, 62.5 mg/ml) of OLE and incubated overnight at 30°C with agitation at 200 rpm. Aliquots (20-μL) of bacterial suspensions were deposited onto 12-mm micro-cover glass slides (Thermo Scientific Portsmouth NH, USA) and after 30 min; the coverslips were immersed into 50 μL of a fixative solution containing 2.5% glutaraldehyde (Electron Microscopy Sciences, Hatfield, PA, USA) for 30 min. The rest of the procedure was as described previously (Liu et al., 2011). Digital images of topographical features of the bacterial samples were collected using a Quanta 200 FEG environmental scanning electron microscope (FEI Co., Inc., Hillsboro, OR, USA) operated in the high vacuum/secondary electron imaging mode at an accelerating voltage of 10 kV and instrumental magnification 50,000X.

### Biofilm Formation Assays

A single colony of *L. monocytogenes* or *S.* Enteritidis was inoculated into 5 ml of BHI broth and incubated at 37°C overnight with agitation at 200 rpm. The bacterial overnight cultures were diluted 100-fold in Modified Welshimer’s Broth (MWB) (HiMedia Laboratories, Mumbai, India) with glucose as the sole carbon source. Diluted bacterial cultures (150 μL) and 50 μL of OLE at different final concentrations (7.81, 15.6 mg/ml) were added to a 96-well PVC microtiter plate previously rinsed with 70% ethanol. Two hundred microliters of MWB (8 wells) was used as a negative control. The 96-well PVC microtiter plate was incubated at 30°C in a humidified container for 48 h. After removal of the medium, the plate was washed five times with distilled water and air dried for 45 min. The plate was stained with 0.1% Crystal Violet for 45 min and, washed five times with distilled water. After 30 min destaining with 200 μL of 95% ethanol, the absorbance at O.D._595 nm_ was measured using a Tecan Safire 2 microplate reader (Tecan Group Ltd., Switzerland). Eight replicates were performed for each sample. All of the absorbance at O.D._595 nm_ was normalized by substracting the media only O.D._595 nm_ numbers. Any absorbance at O.D._595 nm_ above 0 is assumed to indicate some biofilm formation.

### Statistical Analysis

Statistical analysis (student’s *t*-test) was performed using GraphPad InStat software where *p* < 0.05 was considered significant.

## Results

### OLE Inhibited the Growth of Foodborne Pathogens and Reduced Cell Motility in *L. monocytogenes*

**FIGURE 1:**
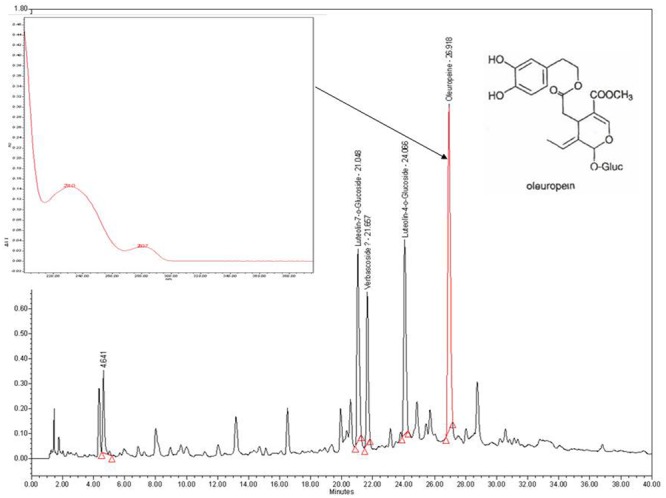
**High performance liquid chromatography (HPLC) chromatogram of polyphenols from olive leaf extract (OLE) using Waters Symmetry C_18_ (5 μm particle) column (3.9 mm × 150 mm) maintained at 35°C.** The flow rate was 1 ml/min and the absorbance changes were monitored at 280 nm.

The polyphenols in OLE were extracted and tested against three foodborne pathogens: *L. monocytogenes* F2365, *S.* Enteritidis, and *E. coli* O157:H7. As shown in **Table 1** and **Supplementary Figure S1**, at a concentration of 62.6 mg/ml, OLE completely inhibited the growth of *L. monocytogenes* and *S.* Enteritidis. The growth of *E. coli* O157:H7 was inhibited by 95%. HPLC was performed to further characterize the phenolic compounds of OLE (**Figure 1**). Four major peaks were identified in OLE: Luteolin-7-*o*-Glucoside, Luteolin-4-*o*-Glucoside, Oleuropein, and Vabascoside. Of the four major peaks, oleuropein represented the major component of olive leaf polyphenols. Oleuropein is the most extensively studied polyphenol in olive trees (Soler-Rivas et al., 2000). It is very abundant in early stage of olive development (Omar, 2010). Oleuropein is known to possess antimicrobial activities (Juven and Henis, 1970; Fleming et al., 1973; Vaughn, 1975). Verbascoside is a conjugated glucoside of hydroxytyrosol and caffeic acid. Commercial oleuropein and verbascoside compounds were also tested against the three foodborne pathogens. At a concentration of 25 mg/ml, oleuropein inhibited the growth of *L. monocytogenes* (94%), *E. coli* O157:H7 (58%), and *S*. Enteritidis (36%). At the same concentration, vabascoside was more effective than oleuropein in inhibiting *L. monocytogenes* (100%), *E. coli O157:H7* (82%), and *S.* Enteritidis (65%) (**Table 1**). Taken together, the crude OLE was less effective in inhibiting the Gram-negative bacteria *E. coli* O157:H7 and *S*. Enteritidis compared to the Gram-positive bacterium *L. monocytogenes*. The commercial compounds oleuropein and vabascoside are more potent than the crude OLE in inhibiting *L. monocytogenes*, confirming that they may be primary components of OLE that cause the inhibition of foodborne pathogens. Both oleuropein and vabascoside were less effective in inhibiting gram negative bacteria *E. coli* O57:H7 and *S.* Enteritidis compared to the Gram-positive bacterium *L. monocytogenes*. This is consistent with the fact that Gram-negative bacteria are less sensitive to polyphenols than Gram positive bacteria (Seow et al., 2014). The Gram-positive bacteria are sensitive to polyphenols since the bacterial membranes interact with hydrophobic components of the polyphenols. On the other hand, Gram-negative bacteria are more resistant to polyphenols because they possess a hydrophilic cell wall (Calo et al., 2015).

**Table 1:** Growth inhibition of OLE to *Escherichia coli O157:H7, Salmonella* Enteritidis, and *Listeria monocytogenes.*

	Bacterial growth inhibition (%)		
	*L. monocytogenes* F2365	*S.* Enteritidis	*E. coli* O157:H7
Olive leaf extract (OLE) (62.5 mg/ml)	100	100	95
Oleuropein (25 mg/ml)	94	36	58
Verbascoside (25 mg/ml)	100	65	82

**FIGURE 2:**
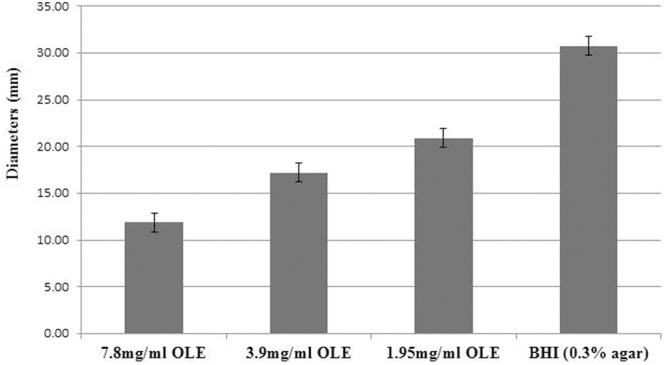
**Motility of *Listeria monocytogenes* F2365 cells in varying concentrations of commercial OLE.** *L. monocytogenes* F2365 strain was stabbed into BHI soft agar (0.3%) and incubated at room temperature for 48 h. The diameters of the bacterial growth rings were measured. Data presented are the averages of nine measurements with standard deviations.

A previous study showed that olive waste extract was able to reduce bacterial motility in *E. coli* (Carraro et al., 2014). To test whether OLE inhibited the cell motility in *L. monocytogenes*, cell motility assays (swimming assays) were performed. As shown in **Figure 2**, *L. monocytogenes* had reduced motility when treated with a sub-lethal dose (1/32X MIC, i.e., 1.95 mg/ml) of OLE, while the motility was further reduced when increased doses of OLE (1/16X and 1/8X MICs) were applied.

### *L. monocytogenes* Showed Loss of Flagella with OLE Treatment

**FIGURE 3:**
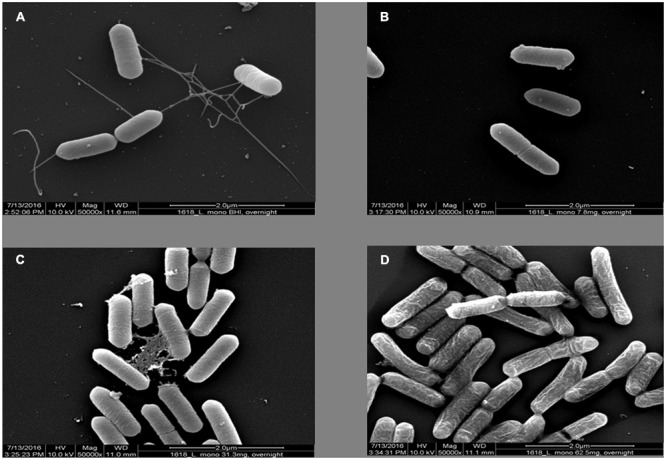
**Scanning Electron Microscopy pictures (magnification 50000×) of *L. monocytogenes* cells in different concentrations of OLE. (A)** *L. monocytogenes* in 0.3% BHI agar plate. **(B)** *L. monocytogenes* in 7.8 mg/ml (1/8XMIC) OLE. **(C)** *L. monocytogenes* in 31.3 mg/ml (1/2XMIC) OLE. **(D)** *L. monocytogenes* in 62.5 mg/ml (MIC) OLE.

Since OLE reduced the motility of *L. monocytogenes*, we tested whether the reduced motility was due to loss of flagella in *L. monocytogenes*. The mode of action of OLE against *L. monocytogenes* was investigated by examining the cell morphology using scanning electron microcopy. As shown in **Figure 3**, *L. monocytogenes* cells displayed normal flagella without OLE treatment, but lost flagella at a sub-lethal dose (1/8X MIC) of OLE (7.8 mg/ml). Obvious cell membrane damage was also observed at the lethal dose (MIC) of 62.5 mg/ml OLE treatment. Hence, the absence of flagella in *L. monocytogenes* appeared to correlate with the observed reduced motility (**Figure 2**).

### OLE Inhibited Biofilm Formation in *L. monocytogenes* and *S.* Enteritidis

**FIGURE 4:**
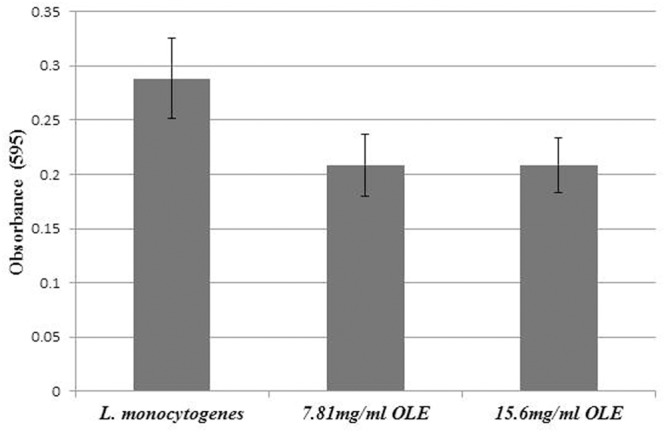
**Biofilm formation of *L. monocytogenes* at different concentrations (1/8X and 1/4X MICs) of OLE.** Assays were performed in PVC microtiter plates by incubating *L. monocytogenes* with different concentrations of OLE in Modified Welshimer’s Broth (MWB) media at 30°C for 48 h. Values are means and standard deviations of eight replicates.

**FIGURE 5:**
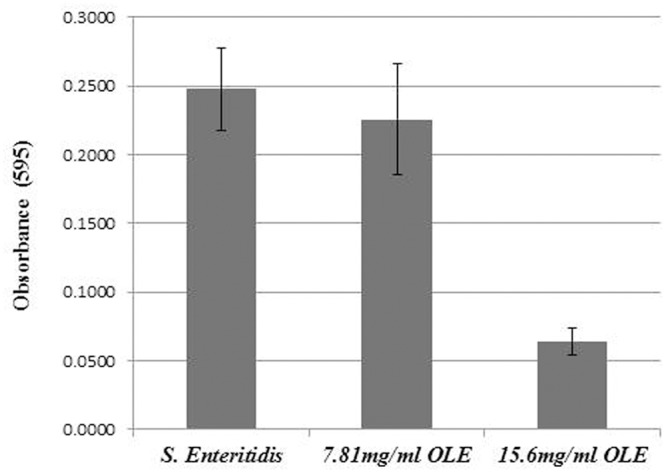
**Biofilm formation of *Salmonella* Enteritidis at different concentrations (1/8X and 1/4X MICs) of OLE.** The biofilm growth was assessed by crystal violet staining and quantified at 595 nm using a spectrophotometer. Values are means and standard deviations of eight replicates.

A previous study showed the olive mill waste was able to reduce biofilm formation in *E. coli* (Carraro et al., 2014). Since OLE abolished flagella, which is crucial for biofilm formation in *L. monocytogenes* (Lemon et al., 2007), we hypothesized that OLE may inhibit biofilm formation. To test this hypothesis, biofilm formation assays were performed using *L. monocytogenes* and *S*. Enteritidis cells. As shown in **Figure 4**, OLE slightly inhibited biofilm formation in *L. monocytogenes* at the concentration of 7.8 mg/ml (1/8XMIC). In *S*. Enteritidis, biofilm formation was inhibited by 74% (100^∗^(0.248-0.064)/0.248 = 74%) at a concentration of 15.6 mg/ml (1/4XMIC) (**Figure 5**). Since biofilm formation is a major problem for the food industry, OLE has the potential to be used in the control bacterial biofilms in food and food-related environments.

## Discussion

Polyphenols are major components in olive leaves. In addition, residual levels of pesticides such as trichlorfon, fenthion and its metabolites can be detected in olive leaves. However, the time gap between treatment and harvest strongly influences the residue levels of pesticides in olives. So, it is necessary to stop using the insecticides 60 days before harvest since they leave low residue levels in oils (Cavanna and Molinari, 1998). Of the four major polyphenol compounds identified in our study, oleuropein and vabascoside were tested for their antimicrobial activities against the three foodborne pathogens. Compared to the crude extract, oleuropein, and vabascoside were more potent for the inactivation of the studied foodborne pathogens (**Table 1**). Although present in olive seeds, oleuropein is present predominantly in olive leaves (Le Tutour and Guedon, 1992). Oleuropein has been used in pharmacology to treat inflammation and in the control of obesity (Vogel et al., 2014). Its application in the food industry is limited due mainly to its bitter taste. The antimicrobial mechanism of oleuropein in *S. aureus* has been studied using a system biology approach. The genome model predicted the changes in gene expression, which correlated very well with experimental data (Li et al., 2016). Vabascoside is another abundant polyphenol present in olive leaves. It is more effective than oleuropein in inhibition of bacterial growth.

Our results also demonstrated that OLE was able to abolish the flagella in *L. monocytogenes*, and thus, to reduce the pathogen’s motility. This theory was confirmed with the utilization of scanning electron microscopy (SEM). We were able to observe the lack of flagella in *L. monocytogenes* grown in the presence of OLE (**Figure 3**). Disruption of the cell surface of *L. monocytogenes* grown in the presence of lethal dose of OLE (62.5 mg/ml) were also observed, indicating that OLE may be having a negative effect on the bacterium, other than abolishing flagella production.

Our results showed that the crude OLE was effective in inhibiting the growth of the three foodborne pathogens, providing support that OLE could potentially be used alone or in conjunction with other antimicrobials for the purpose of pathogen control in food products. The MIC (62.5 mg/ml) of OLE for *L. monocytogenes* and *S.* Enteritidis was somewhat high compared to that of synthetic antimicrobials; however, it can be used in combination with other hurdle technologies such as heat and acid treatments. The combination of two or more antimicrobial intervention treatments in lower dose may act in a synergistic manner. For example, the combined application of olive powder with high hydrostatics pressure resulted in the inactivation of *Bacillus cereus* spores (Marco et al., 2011). In another study, OLE increased the efficacy of ampicillin *in vitro* (Lim et al., 2016). In our preliminary study, OLE incorporated into packaging film was able to inhibit the growth of *E. coli* K12 and *Listeria innocua* (Jin, et al., unpublished data).

The present work provides a baseline in the study of the antimicrobial properties and mechanism of action of OLE. However, additional research needs that should be addressed in the future experiments involve the conductance of transcriptomic (RNA-seq) analyses on *L. monocytogenes* treated with sub-lethal doses of OLE to identify gene expression changes with exposure to OLE. Since OLE was able to abolish flagella and reduce the motility of *L. monocytogenes*, we hypothesize that genes involved in synthesis of flagella and motility may be altered by OLE treatments. Thus, OLE can potentially be used as a part of multiple hurdle intervention approach to control pathogens in foods. Further studies will explore the use of lower concentrations of OLE in combination with other treatments, including treatment with lactic acid and other antimicrobial compounds. Overall, a better understanding of how pathogens respond to OLE treatment is expected to contribute to the optimization of preservation strategies aiming at the effective control of foodborne pathogens.

## Author Contributions

YL designed the experiments and wrote the manuscript. NM helped design and perform the experiment related to extraction and analysis of OLE. LM conducted the experiments. Each author substantially contributed to the work reported here.

## Conflict of Interest Statement

The authors declare that the research was conducted in the absence of any commercial or financial relationships that could be construed as a potential conflict of interest.

## Footnotes

1 http://www.cdc.gov/foodborneburden/2011-foodborne-estimates.html

## Supplementary Material

FIGURE S1 **Growth of *Salmonella* Enteritidis over a 24 h period, in the presence of different concentrations of olive leaf extract (OLE)**.
