# Supplementary figures and images for: Assessment of the Antimicrobial Activity of Olive Leaf Extract Against Foodborne Bacterial Pathogens
Source: Front Microbiol. 2017 Feb 2;8:113. doi: 10.3389/fmicb.2017.00113 (PMC5288333; doi:10.3389/fmicb.2017.00113)

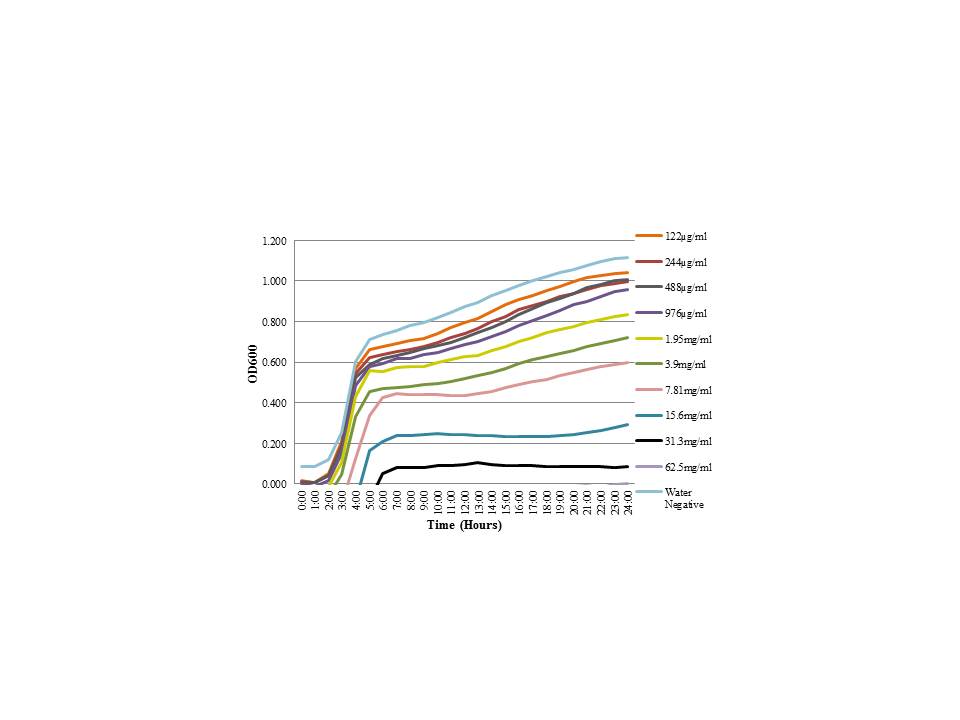

Supplement: FIGURE S1 — Growth of Salmonella Enteritidis over a 24 h period, in the presence of different concentrations of olive leaf extract (OLE). [file Image_1.JPEG]
